# The Drinking Effect of Hydrogen Water on Atopic Dermatitis Induced by *Dermatophagoides farinae* Allergen in NC/Nga Mice

**DOI:** 10.1155/2013/538673

**Published:** 2013-11-20

**Authors:** Rosa Mistica C. Ignacio, Hyun-Suk Kwak, Young-Uk Yun, Ma. Easter Joy V. Sajo, Yang-Suk Yoon, Cheol-Su Kim, Soo-Ki Kim, Kyu-Jae Lee

**Affiliations:** ^1^Department of Environmental Medical Biology, Wonju College of Medicine, Yonsei University, Wonju, Gangwon 220-701, Republic of Korea; ^2^ECO Solution Team, Digital Media and Communications R&D Center, Samsung Electronics Co., Ltd., Suwon, Gyeonggi 443-742, Republic of Korea; ^3^Department of Microbiology, Wonju College of Medicine, Yonsei University, Wonju, Gangwon 220-701, Republic of Korea

## Abstract

Hydrogen water (HW) produced by electrolysis of water has characteristics of extremely low oxidation-reduction potential (ORP) value and high dissolved hydrogen (DH). It has been proved to have various beneficial effects including antioxidant and anti-inflammatory effects; however, HW effect on atopic dermatitis (AD), an inflammatory skin disorder, is poorly documented. In the present study, we examined the immunological effect of drinking HW on *Dermatophagoides farinae*-induced AD-like skin in NC/Nga mice. Mice were administered with HW and purified water (PW) for 25 days. We evaluated the serum concentration of pro-inflammatory (TNF-**α**), Th1 (IFN-**γ**, IL-2, and IL-12p70), Th2 (IL-4, IL-5, and IL-10), and cytokine expressed by both subsets (GM-CSF) to assess their possible relationship to the severity of AD. The serum levels of cytokines such as IL-10, TNF-**α**, IL-12p70, and GM-CSF of mice administered with HW was significantly reduced as compared to PW group. The results suggest that HW affects allergic contact dermatitis through modulation of Th1 and Th2 responses in NC/Nga mice. This is the first note on the drinking effect of HW on AD, clinically implying a promising potential remedy for treatment of AD.

## 1. Introduction

Atopic dermatitis (AD) is an allergic inflammatory skin disorder characterized by impaired immunological responses [1]. AD usually affects 10% to 20% infants and young children, but it can persist into adulthood (1%–3%) since it is often a long-lasting skin disease [2]. AD has a complex etiology involving interrelationship of several factors such as genetic, environmental, pharmacologic, psychological, immunologic and skin barrier dysfunction [3]. The altered immune function received special attention as the major factor contributing to the onset, development, and severity of AD. AD is well characterized by having clinical phenotype such as elevated serum IgE levels, peripheral eosinophilia, and eczematous skin lesions infiltrated by inflammatory cells [4, 5]. NC/Nga mice were the first representative animal model for investigating and developing treatment on AD-like skin disease [6, 7]. In conventional surroundings, NC/Nga mice were observed to spontaneously develop skin lesions characterized by scratching behavior, erythema and hemorrhage, edema, scaling, and dryness of the skin comparable to human AD [6]. Under pathogenic-free surrounding, NC/Nga mouse model does not show skin lesions and thus AD-like symptoms are triggered by exposure to a stimulus. *Dermatophagoides farinae* (Df) is a cosmopolitan species of house dust mites and a common contributory cause of AD. The topical application of this allergen Df extract (DfE) produces atopic dermatitis-like skin lesions in NC/Nga mice [8, 9]. Therefore, we adopted NC/Nga mouse model exposed with DfE mimicking the pathogenesis of human AD. 

 Hydrogen molecule has been demonstrated to have excellent antioxidant as well as anti-inflammatory properties. Active hydrogen acts as antioxidant and showed protection of oxidative-induced damage *in vitro* [10]. It has also been reported that it can selectively scavenge reactive oxygen species (ROS) [11] and showed positive influence in cytokine imbalance [12]. Hydrogen is known to easily penetrate the skin, diffuses rapidly into tissues and cells, and distributes in the body through blood flow [13]. H_2_ can be incorporated in the body through inhalation of H_2_ gas, drinking water with dissolved H_2_, and injecting saline with dissolved H_2_. HW produced by electrolysis of water is included of the drinking water containing high concentration of hydrogen gas and has properties of high dissolved hydrogen (DH), negative oxidation-reduction potential (ORP) level, and neutral pH. Previous studies on electrolyzed reduced water (ERW) and alkaline reduced water, which are also hydrogen-rich water produced by the same reaction with HW, showed various beneficial effects such as reducing the levels of serum triglyceride and blood glucose in OLEFT diabetic rat model [14, 15], prevention of insulin resistance [16], and liver inflammation [17]. In addition, the oral intake of hydrogen-rich water showed antiallergic effect *in vivo* [18]. However, at present, there is no evidence that drinking water rich in DH provides benefits for patients with AD. Thus, the objective of the study was to examine the drinking effect of hydrogen water (HW) with high DH, low ORP, and neutral pH on AD induced by repeated application with DfE ointment in NC/Nga mice. We might expect drinking HW to complement the topical drugs for AD through modulating the Th1 and Th2 responses.

## 2. Materials and Methods

### 2.1. Preparation of Control Water (Purified Water)

Purified water (PW) was prepared by using tap water (TW) as source water. The TW was purified by reverse osmosis (RO) process (R/O Membrane Filter, Coway Co. Ltd., Gumi, Republic of Korea). In the RO process, the TW is push against a special semipermeable membrane from the pressure on the waterline that causes the molecular squeezing thereby separating the water molecules from the contaminants. The water molecules then pass through to the inside of the membrane. The R/O Membrane Filter rejects contaminants based on their size and it is equipped with an ultrafiltration pore that has a size around 0.001 *μ*m. PW generated was used as a control water given to positive control (PC) group and it had an ORP value of 292 ± 5 mV, DH of 0.002 ± 0.001 ppm, and a pH of 7.01 ± 0.02.

### 2.2. Preparation of Test Water (Hydrogen Water)

HW was prepared by using PW generated through RO and ultrafiltration. The PW was further processed by water-electrolyzing apparatus (Samsung Highly Reactive Hydrogen Reduced Water Maker, Samsung Electronics Co., Ltd., Korea) and was collected from water tank containing cathode platinum plate. ORP value was controlled to −510 ± 6 mV (HM-21P, TOA Electronics Co., Japan), and DH value was controlled to 0.50 ± 0.02 ppm (DH-35A, DKK-TOA Co., Japan) through electrolysis of water. pH of HW was regulated to 7.30 ± 0.02 through neutralization of OH^−^ generated during electrolysis in cathode water tank. HW generated was used as a test water given to HW group.

### 2.3. Animals

Four-week-old male NC/Nga mice weighing 25 ± 2 g were purchased from Orient Bio Inc. (Korea) and were maintained for 1 week prior to experiments. Mice were housed in stainless steel cages in a controlled environment with temperature of 22 ± 2°C and 40–60% humidity under a 12 : 12-hour light-dark cycle. The Animal Use and Care Protocol for this Animal Experiment was approved by the Institutional Animal Care and Use Committee (IACUC) at Wonju Campus, Yonsei University, Gangwon, Wonju, Republic of Korea.

### 2.4. Induction of Atopic Dermatitis in NC/Nga Mice

 AD-like skin lesions in NC/Nga mice were performed using mite antigen as described previously [19]. The hair on the back was shaved with depilatory cream (Veet, Oxy Reckitt Benckiser Ltd., France) 1 day before experiments. The exposed dorsal region was treated with 200 *μ*L with DfE ointment (Biostir-AD, Biostir, Kobe, Japan) prepared from house dust mites, a crude extract allergen of *Dermatophagoides farinae* as described previously [9], twice a week for 3 weeks. NC/Nga mice were randomly assigned to two groups: PW (*n* = 12) and HW (*n* = 12). Mice received the last water treatment (water administration) on day 25 and were sacrificed on day 26 to evaluate immunological changes.

### 2.5. Analysis of Th1, Th2, and Proinflammatory Cytokines

Cytokines secreted in the blood were investigated. Serum concentrations of IL-2, IL-4, IL-5, IL-10, IL-12p70, GM-CSF, TNF-*α*, and IFN-*γ* were measured using Multiplex kit (Bio-Rad, San Diego, USA) and run on Luminex technology (Bio-Plex Multiplex Bead Array System, Bio-Rad Hercules, CA, USA) according to the manufacturer's instruction. Raw fluorescence data were analyzed by the software using a 5-parameter logistic method. 

### 2.6. Statistical Analysis

Data values were expressed as the mean ± SEM. The mean values among groups were analyzed and compared by one-way analysis of variance followed by subsequent multiple comparison test (Tukey) with Graph Prism version 5.0 software packages (GraphPad Software, USA). Differences were considered statistically significant at *P* < 0.05, *P* < 0.01, and *P* < 0.001.

## 3. Results and Discussion

**Figure 1:**
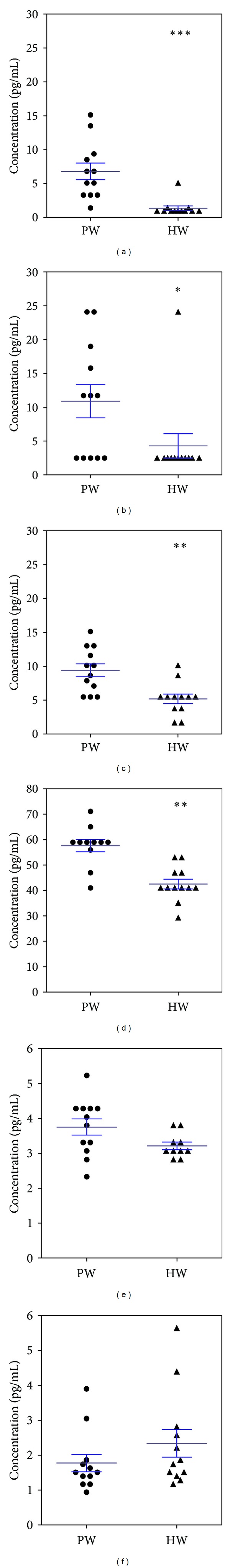
Effect of drinking hydrogen water (HW) on proinflammatory, T helper (Th) 1, and Th2 cytokines. Mice administered with HW significantly decreased *Dermatophargoides farinae* extract had (DfE-)induced cytokines IL-10 (a), GM-CSF (b), IL-12p70 (c) and TNF-*α* (d) levels. IL-2 (e) and IL-4 (f) did not differ significantly between HW and purified water (PW) groups. Data are mean ± SD, *n* = 12. *P < 0.05, **P < 0.01, and ***P < 0.001 indicate significant differences tested with ANOVA. Tukey's test was used for *post hoc* tests.

At present, there is no evidence for the drinking effect of HW on AD. Here, we investigated the relationship between drinking HW and AD through examination of various cytokines closely related to AD and examined therapeutic possibility of HW. Repeated application of DfE to NC/Nga mice leads to atopic dermatitis-like skin lesions characterized by impaired immune response. Previous studies have pointed out the importance of Th1 and Th2 cytokines, which are found to be overexpressed on the skin as means of inflammatory response on experimental models of allergen-induced allergic inflammation in mice [5]. Moreover, after the DfE application, Th1, Th2, and proinflammatory cytokines were increased in concentration as demonstrated by the study of Sung and colleagues [20]. Here, we investigated the effect of drinking HW on serum cytokines (Th1, Th2 and pro-inflammatory) using Luminex-bead array system known for its high sensitivity and precision. The supplementation of HW significantly decreased DfE-induced cytokines IL-10 (Figure 1(a)), GM-CSF (Figure 1(b)), IL-12p70 (Figure 1(c)), and TNF-*α* (Figure 1(d)) levels. IL-10 produced mainly by Treg cells participates in the immune dysregulation that is characteristic of human AD and IL-10 is suggested that it inhibits cell-mediated immunity, which may account for the reduced allergic contact reactivity in AD cases [21]. IL-10, which is one of the traditional cytokines involved in AD, has been regarded to have a conflicting role. Production of IL-10 by CD4+ T cells varies depending on the severity of AD, fewer on severe AD as compared to mild AD [22]. Thorough understanding of the effect of IL-10 will help in the interpretation of the pathogenesis of AD. As noted by Niwa [23]; plasma levels of IL-10 seem to correlate inversely with the severity of AD. However, increased production of IL-10 has been demonstrated by other studies [21, 24]. T cells stimulated with IL-12 showed induction of IL-10 *in vitro* [25]. The lower level of IL-10 in our study might be correlated with the decreased level of IL-12p70. IL-12, which consists of p35 and p40 subunits, enhances Th1 cytokine production [26]. These results suggest that HW suppresses the development of AD by suppressing the cytokines level. In contrast, levels of IL-2 (Figure 1(e)) and IL-4 (Figure 1(f)) did not differ significantly between HW and PW (control) groups. Additionally, IL-5 and IFN-*γ* were undetectable in the serum of NC/Nga mice (data not shown). These results could be, in part, explained by the variability in the source of the samples (blood or skin), the time the sample was collected, and also by the different assay methods employed. 

 The repeated application of the allergen induced the increased production of Th2 cytokines [27]. The supplementation of HW suppressed this cytokine production. The chemical properties of HW that we used in this study are high DH, low ORP, and neutral pH. The abrogating effect of HW on Th1, Th2, and pro-inflammatory cytokines in AD model is attributable to the rich hydrogen content of HW. As discussed by Xie et al. [28], hydrogen gas has anti-inflammatory properties in zymosan-induced generalized inflammation model partly by reducing the levels of pro-inflammatory cytokines in the serum. In our previous studies related to immunological effect of hydrogen-rich ERW, bathing was found to restore cytokine imbalance in mice model with induced UVB skin injury [12] and drinking ERW ameliorated obesity through restoring adipokine and inflammatory cytokines network [29]. Given these reported data, the beneficial effect of DH in HW might be due, in part, to the anti-inflammatory property of hydrogen [30], such as the downregulation of Th1, Th2, and pro-inflammatory cytokines. 

 Hydrogen represents an emerging safe and potent medical gas that promises to provide a treatment and preventive control of various diseases. Hydrogen can be administered into the body through drinking water rich in H_2_ such as HW, inhaling H_2_ gas, injecting saline with dissolved H_2_, and taking a hydrogen bath. Among these accepted methods of administration of hydrogen in the body, drinking or oral intake of hydrogen-rich water is the most convenient and suitable for continuous consumption for therapeutic use. Not only hydrogen has anti-inflammatory effects, but also several studies had demonstrated that hydrogen exhibits antioxidative stress effects [11, 13, 30, 31]. Of note, H_2_ has several advantages as potential antioxidant since it is mild enough not to disturb any metabolic oxidation-reduction reactions, thus sparing the mammalian cells in initiating defense mechanism leading to the production of ROS [11]. Our HW might be a feasible candidate as antioxidant not only that it has a high DH, also it has low (negative) ORP. “As discussed elsewhere [32]”; decreased ORP means increased reducing capacity. In the water ionizer industry, ORP is equivalent to antioxidant potential, and the negative implies the reduction power of the water. This study is the first to provide evidence for the drinking effect of HW on AD. To uncover the profound mechanism of HW on inflammatory response, further studies targeting signaling pathways and ROS production would be necessary, as AD is a type of oxidative stress-related and inflammatory disease. This study opens a new insight into the new therapeutic and preventive treatment of AD, applying safer fluid such as HW. In addition, our results give attention and advance understanding to those traditional cytokines that could possibly affect the severity of AD. Together these data, although conflicting, suggest that the role of traditional cytokines involved in AD needs to be examined scrupulously and further studies on how HW works on molecular level still await an answer. Lastly, the potential of HW as antioxidant is underway.

## 4. Conclusions

To conclude, drinking HW suppressed the levels of inflammation-related mediators such as Th1, Th2, and pro-inflammatory cytokines which are number one players in the pathogenesis of human AD. In addition, this study provides a new insight of the relevance of certain cytokines in AD. HW represents a potentially alternative therapeutic and preventive treatment of AD.

## Abbreviations

H_2_: Molecular hydrogen
AD: Atopic dermatitis
HW: Hydrogen water
Df: *Dermatophargoides farinae*
DfE: Df extract
ORP: Oxidation reduction potential
DH: Dissolved hydrogen
PW: Purified water
TW: Tap water
RO: Reverse osmosis
IACUC: Institutional Animal Care and Use Committee
ERW: Electrolyzed reduced water
ROS: Reactive oxygen species.
